# The centennial of the pecking order: current state and future prospects for the study of dominance hierarchies

**DOI:** 10.1098/rstb.2020.0432

**Published:** 2022-02-28

**Authors:** Eli D. Strauss, James P. Curley, Daizaburo Shizuka, Elizabeth A. Hobson

**Affiliations:** ^1^ Department of Collective Behavior, Max Planck Institute of Animal Behavior, Konstanz, Germany; ^2^ Centre for the Advanced Study of Collective Behaviour, University of Konstanz, Konstanz, Germany; ^3^ School of Biological Sciences, University of Nebraska Lincoln, Lincoln, NE, USA; ^4^ Department of Psychology, University of Texas at Austin, Austin, TX, USA; ^5^ Department of Biological Sciences, University of Cincinnati, Cincinnati, OH, USA

**Keywords:** social status, social evolution, immunology, aggression, rank, social cognition

## Abstract

A century ago, foundational work by Thorleif Schjelderup-Ebbe described a ‘pecking order’ in chicken societies, where individuals could be ordered according to their ability to exert their influence over their group-mates. Now known as dominance hierarchies, these structures have been shown to influence a plethora of individual characteristics and outcomes, situating dominance research as a pillar of the study of modern social ecology and evolution. Here, we first review some of the major questions that have been answered about dominance hierarchies in the last 100 years. Next, we introduce the contributions to this theme issue and summarize how they provide ongoing insight in the epistemology, physiology and neurobiology, hierarchical structure, and dynamics of dominance. These contributions employ the full range of research approaches available to modern biologists. Cross-cutting themes emerging from these contributions include a focus on cognitive underpinnings of dominance, the application of network-analytical approaches, and the utility of experimental rank manipulations for revealing causal relationships. Reflection on the last 100 years of dominance research reveals how Schjelderup-Ebbe's early ideas and the subsequent research helped drive a shift from an essentialist view of species characteristics to the modern recognition of rich inter-individual variation in social, behavioural and physiological phenotypes.

This article is part of the theme issue ‘The centennial of the pecking order: current state and future prospects for the study of dominance hierarchies’.

## 1. Introduction

Anyone who thinks the inhabitants of a chicken yard are thoughtless happy creatures with a daily life of undisturbed pleasure … is thoroughly mistaken. A grave seriousness lies over the chicken yard

—Schjelderup-Ebbe 1922 [1, p. 39]

At the age of ten, Thorleif Schjelderup-Ebbe's fascination with domestic chickens led him to collect systematic data on patterns in the aggressive interactions among individual chickens [2]. At this young age, he saw past the chickens' outwardly similar appearance and mannerisms, and by learning to identify each individual, he became captivated by the differences in behaviour among them. He noticed that although the chickens frequently pecked each other during feeding competition, not all chickens were pecked equally. Instead, certain hens were the ‘despots’, able to peck all others, whereas other hens were pecked by all others. In fact, through careful observation of which hens were pecking which, Schjelderup-Ebbe found that members of his chicken societies were highly variable in their tendency to peck and be pecked, and could be arranged in order of their ability to peck others. In 1922, years later, he published these observations from his childhood data notebooks as his enduring work [1] describing a ‘Hackliste’, translated to English as a ‘pecking order’ [3], and now known in academic circles as a dominance hierarchy.

In the decades following Schjelderup-Ebbe's foundational papers [1,4], publications on dominance hierarchies increased dramatically, and although growth has fluctuated over time (see Hobson [5] in this theme issue), research on dominance hierarchies has expanded over the last century to become a central concept in behavioural ecology. Now, dominance hierarchies appear frequently in the contemporary study of animal physiology [6], immunology [7], cognition [8], animal welfare and husbandry [9], cooperative breeding and reproductive skew [10], human health and ageing [11], modern human behaviour [12], and human evolution [13].

This theme issue marks the 100th birthday of the concept of a dominance hierarchy. In this introduction, we briefly summarize the features of dominance hierarchies that have been well established over the last 100 years, then highlight the emerging topics that will drive the next century of new insights into this fundamental social structure.

## 2. What have we learned in the last century?

Although many lines of inquiry continue to produce new insights into dominance hierarchies, some questions have been satisfactorily answered during the last century. To serve as a roadmap for new discoveries about dominance as a structuring feature of societies, the contents of this theme issue deal primarily with ongoing and emerging areas of insight. However, to acknowledge the century of high-quality research upon which these new insights rest, we here briefly discuss the pillars of established knowledge generated over the past century of dominance hierarchy research.

### (a) Are dominance hierarchies found in many species?

Despotism is the basic idea of the world, indissolubly bound up with all life and existence

—Schjelderup-Ebbe, according to Allee 1939 [14, p. 185]

**Nearly all societies are structured by some type of dominance hierarchy**. Schjelderup-Ebbe's observation of hierarchically structured aggression in chickens prompted research aimed at understanding potentially similar hierarchical structures in other species. This early work focused primarily on the societies of other species of birds [14], primates [15] and domestic animals [16]. The mathematical foundations for the measurements of hierarchies were also presented around this time by Kendall & Smith [17] and Landau [18,19]. Considerable research has now clearly demonstrated that these dominance hierarchies are a widespread feature of societies occurring broadly across the evolutionary tree ([20,21]; see also Strauss *et al*. [22] in this theme issue) including humans (see Redhead & Power [23] and Zeng *et al.* [24] in this theme issue).

### (b) Is dominance a feature of individuals, relationships or groups?

There are no two individual birds of any given species which, when living together, do not know which of the two has precedence and which is subordinate

—Schjelderup-Ebbe 1935 [4, p. 949]

**Dominance hierarchies are networks of dominance relationships, which are determined by asymmetrical displays of threat and, especially, subordination**. Research into dominance hierarchies underwent a pubertal phase of awkward self-realization during the mid-twentieth century, brought on by multiple factors. At this time, primatologists were applying Schjelderup-Ebbe's concept of a pecking order in the societies of wild and captive primates, many of which lacked intuitive and unambiguous dominance interaction signals such as pecking/fleeing and had dominance hierarchies that were not as obvious and linear as those found in chickens. During these studies, variation in access to food and mating success were often mistakenly used as proxies for dominance, leading to hand-wringing when studies began to reveal that aggression, copulation and access to food were often not strongly correlated [25]. Concurrently, conceptual confusion appeared in the literature regarding whether dominance was most appropriately viewed as a feature of individuals or a relational property [26–28]. These factors led to several debates about the utility of dominance hierarchies as a concept ([25,28] and commentary [28]), with Robert A. Hinde likening dominance hierarchy research to a conceptual ‘strait-jacket’ [26, p. 1] and ‘groan[ing] at the sight of another article on the overused, often misused, over-discussed but nevertheless often useful concept of dominance’ [29, p. 442].

Despite these growing pains, the field of dominance hierarchy research emerged with a general consensus on the nature of dominance that has provided a robust foundation for a mature study of dominance hierarchies. In a thorough review of the debate and the various proposed definitions, Drews [30, p. 308] synthesized a concept of dominance that forms the basis of modern understanding of dominance hierarchies as an organizing structure in animal societies: ‘Dominance is an attribute of the pattern of repeated, agonistic interactions between two individuals, characterized by a consistent outcome in favour of the same dyad member and a default yielding response of its opponent rather than escalation’. Dominance therefore describes a dyadic social relationship [26] that emerges from sequences of agonistic interactions [31] where one individual exhibits subordination [32]. Following from this definition, dominance hierarchies are the group-level structure arising from the social network of these dyadic relationships, and although dominance is not a feature of individuals, dominance rank can be a useful measure for summarizing an individual's position in this network (see McCowan *et al.* [33] and Redhead & Power [23] in this theme issue).

### (c) Are dominance hierarchies variable in structure?

One might distinguish mild and strict despotism … . These vary in degree, so that we have many kinds of despots, from the one which shows great reasonableness in his practice towards another to the one that acts as the most cruel tyrant

—Schjelderup-Ebbe 1935 [4, p. 950]

**Dominance hierarchies show structural variability along multiple dimensions, but are consistently more transitive than expected by chance**. Research into dominance hierarchies across species has revealed considerable variation in hierarchy structure. One of the most discussed dimensions of structural variability is linearity, or the extent to which patterns of the outcomes of agonistic interactions conform to a perfect rank order where each individual defeats all (and only) individuals ranked below them [18,19]. A related concept is transitivity, or the tendency for triadic motifs to have transitive properties (i.e. A → B and B → C implies A → C) [34,35]. Dominance hierarchies are also made up of dyadic relationships that can vary in directional consistency, or the consistency with which the higher-ranked member of the dyad is observed to win interactions [36] (e.g. consider a dyad where the dominant individual wins 51% of interactions versus one where it wins 100% of interactions). Finally, dominance hierarchies can also vary in steepness, or the degree to which the propensity to win changes with rank (e.g. ‘egalitarian’ versus ‘despotic’ hierarchies) [37,38].

Many of these measures of hierarchy structure are biased by the number of missing observations (i.e. dyads for which there are no interaction data) [39], making it difficult to derive insight from cross-species comparisons of hierarchy structure (but see [40–42]). However, insofar as it is robust to variation in observed data, network-based methods provide evidence that the tendency towards transitivity is a fundamental organizing principle underlying dominance relationships. For example, propensity towards transitive relationships can be measured by focusing on triads (sets of three individuals) that have been observed to interact (the triangle transitivity measure [35]), or by measuring the frequencies of different ‘network motifs’—i.e. substructures of three- or four-player configurations within the entire dominance network [21,43]. These comparative data show that, despite variation in the structure of dominance hierarchies across taxa, hierarchies are consistently more transitive than expected by chance, showing enrichment for transitive triads and fewer intransitive triads than expected. These results mirror parallel work on hierarchy formation that indicates that this tendency towards transitivity emerges and persists from the start of novel hierarchy formation in experimental groups [34,44].

### (d) What determines dominance rank?

Frequently, momentary courage or luck is decisive in establishing dominance relationships

—Schjelderup-Ebbe 1922 [1, p. 40]

**Position in the dominance hierarchy is determined by a combination of attributes of individuals, stochastic processes, and social context**. In Schjelderup-Ebbe's initial work in chickens [1,4], he noted that although strength was a relevant factor in determining which of a pair of birds is dominant, contextual or stochastic processes also played a role in dominance relationship formation. Since his initial observations, considerable evidence has supported the idea that multiple factors explain position in the dominance hierarchy. Many studies aimed at understanding the determinants of dominance have found significant predictors of dominance—for instance, physical fighting characteristics such as body size in male elephant seals [45], physiological correlates like testosterone secretion in birds [46], status badges such as facial markings in paper wasps [47], or conventions such as maternal rank inheritance in spotted hyenas and many Old World primates [48]. However, mathematical and empirical evidence suggests that these factors are unlikely to fully explain the formation of linear hierarchies [18,44,49]. Considerable evidence demonstrates the existence of winner- and loser-effects, where experiencing a win (loss) leads an individual to increase (decrease) its probability of winning (losing) a future encounter [50–52]. These processes can contribute to hierarchical structure [50], but may not be sufficient to produce the linear hierarchies observed in many animals [53]. Finally, it is clear that social context plays an active role during hierarchy formation; individuals are influenced by the occurrence of interactions among their group-mates, producing more transitive and linear hierarchies than are assembled in the absence of social information [54,55]. In sum, although there is room for more insight into the relative contributions of these different factors (e.g. [56]; see also Tibbetts *et al*. [57] in this theme issue), the evidence conclusively suggests that position in the dominance hierarchy is influenced by a combination of differences among individuals, stochastic processes and social context.

### (e) Does high dominance rank confer advantages?

Those which are despots over many thrive, become stout, look contented; those in the middle rank are usually normal; those which have nearly all the others over them are thin, restless, and often pine away

—Schjelderup-Ebbe 1935 [4, p. 966]

**Position in the dominance hierarchy is often—but not always—associated with fitness-related outcomes**. From the earliest observations of dominance hierarchies, there have been obvious advantages to occupying high dominance status in many species, with dominant individuals often enjoying priority access to food, more reproductive opportunities and greater survival [15]. However, as studies of dominance expanded to more species, the link between dominance status and fitness outcomes began to receive some more sceptical scrutiny [32]. Systematic reviews revealed that high dominance status is typically associated with increased reproductive success, although there is variation in this pattern [20,31]. Nevertheless, the process of this reevaluation of the benefits of dominance status added considerable nuance to our understanding of how dominance relates to individual outcomes. Socio-ecological variables, like dispersal patterns [58,59], the distribution and synchrony of receptive mates [60,61], or abilities for subordinates to leave the group [10], can modulate the strength of the relationship between dominance and individual outcomes. Furthermore, in some cases, achieving and maintaining high dominance status can be costly [62–64]. The relationship between dominance and individual outcomes is also sensitive to the frequency of changes in the hierarchy and can vary over time with shifts in social dynamics or individual behavioural tendencies [65,66]. Finally, ongoing work on the influence of social gradients on health continues to shed new light on the specific mechanisms linking dominance status to individual outcomes ([7,11]; see also Anderson *et al*. [67] and Simons *et al*. [68] in this theme issue).

## 3. What are we still learning?

Having reviewed these ‘answered questions’ in dominance hierarchy research, we now turn to areas of emerging and ongoing research that promise to deliver new insights into the structure, function and evolution of dominance across animal societies.

### (a) Epistemology of dominance

There exists among birds a definite order of precedence or social distinction. The precedence in rank proved to be founded upon certain conditions of despotism

—Schjelderup-Ebbe 1935 [4, p. 949]

The last century has seen continual refinements in our understanding of what dominance is and how we study it. To start this theme issue, contributions explore this history and present modern developments in the study of dominance. Hobson [5] used a science of science approach to determine how the use of terms in titles associated with dominance hierarchy research has changed over the history of the field. This work shows that while the field has moved past the phase of explosive growth in publication rate, we may now be entering a new phase of cross-disciplinary work that blurs the traditional conceptual boundaries. For example, with the coalescence of a definition of dominance grounded in asymmetrical outcomes of agonistic interactions [30], questions arise about how dominance relates to other forms of social power. Lewis [69] discusses these issues in detail, arguing that focusing only on strength, aggression and fighting gives an incomplete picture of the social power dynamics of individuals in hierarchies. Instead, the author presents a power framework that incorporates multiple sources of asymmetry among individuals, emphasizing the importance of ‘leverage’, or asymmetry that arises from control over a resource that cannot be taken by force. The author calls for studies to expand from a focus on aggression-based dominance to a multi-dimensional landscape of power. Given alternative forms of social power in animal systems, it is natural to ask to what extent dominance versus other forms of social power operates in humans. Zeng *et al.* [24] explore the theoretical challenges to applying dominance frameworks to human societies and describe human-specific forms of social dominance. Mirroring the distinction between dominance and leverage discussed by Lewis [69], these authors review research on ‘prestige’, which describes asymmetries in cultural information such as knowledge or skill and offers a non-coercive route to social power. They further detail the empirical evidence supporting a profound role of dominance and prestige as coexisting pathways to individual fitness and social influence in human social groups. For a complementary view of dominance in humans focusing on the socio-relational nature of status hierarchies, see Redhead & Power [23] in this theme issue, under §3c. Finally, the spread of dominance research over the last century has produced a bounty of data on dominance interactions across species, providing the opportunity for comparative research into the evolution of this widespread social structure [21,40–42]. To facilitate future comparative approaches to dominance hierarchy research, Strauss *et al*. [22] collect published dominance interaction data from the last century and share this archive in a freely available R package, *DomArchive*. The authors also reflect on best practices for sharing dominance interaction data moving forward to empower continued comparative insight founded on principles of open science.

### (b) Physiology and neurobiology of dominance

The face of the despot would radiate with joy of satisfied pecking-lust and the fury could clearly be observed in its eyes

—Schjelderup-Ebbe 1935 [4, p. 951]

A major area of ongoing advancement in dominance research is understanding how dominance hierarchies relate to individual physiological and neurobiological states. In this section of the theme issue, contributions take steps towards clarifying the multiple ways in which individual physiology and neurobiology reflect dominance. First, a number of papers review associations between individual state and dominance status. Milewski *et al*. [70] broadly review the suite of behavioural, neural and physiological phenotypic changes that occur as vertebrates attain social dominance or subordinate status. These physiological differences are viewed as plastic responses to the different demands of dominant and subordinate status, although both dominants and subordinates can suffer long-term costs as a result of these modifications. Turning to the societies of invertebrates, Shimoji & Dobata [71] review the development and evolution of dominance hierarchies in eusocial insects, and describe how dominance hierarchies and associated reproductive division of labour emerge through behavioural and physiological suppression of ovarian development in subordinates. Self-organizing processes are implicated in the transition from local behavioural interactions to colony-level dominance hierarchies. Dwortz *et al*. [72] provide insight into how dominance is represented in the brain. In addition to exploring the neural response to sensory signals of dominance status, the authors discuss the role of learning in dominance behaviour and how social memory and associated brain regions serve as cognitive mechanisms for tracking dominance relationships. Accordingly, they identify numerous brain regions that respond to status signals or to the social rank of a familiar conspecific and describe how these pathways are potentially integrated.

This section of the theme issue also contains empirical papers that explore in detail the physiological and neurobiological differences among dominants and subordinates. In a long-term study of wild baboons, Anderson *et al*. [67] find contrasting effects of dominance rank on white blood cell gene expression in males and females, such that high-ranking males resemble low-ranking females. Furthermore, they find that while rank predicted baseline immune gene activity, social bond strength and not rank explained response to immune challenge. Their results highlight how effects of the social landscape can vary based on attributes of individuals (sex) and the specific outcome under investigation. Simons *et al*. [68] came to similar conclusions in an experimental manipulation of rank in captive rhesus macaques. They find that summaries of the occurrence of rank-associated agonistic and affiliative behaviours were often better predictors of specific outcomes than rank itself, but that these relationships varied across outcome and interaction type. They conclude that examining specific social interactions is best for revealing the proximal drivers of social influences on downstream outcomes, but that rank serves as a useful intervening variable [29] for summarizing the overall effects of the social environment on individuals. Finally, to complement these approaches that examine physiological correlates of dominance over extended time-scales, Knoch *et al*. [73] use infrared thermography to measure head temperature phenotypes in response to individual interactions. They find that both exhibiting and receiving aggression was associated with rapid and nonlinear fluctuations in head temperature. These fluctuations in temperature reflect stress-induced hyperthermia in response to a social challenge and shed light on how single interactions evoke the changes that produce socially mediated physiological differences over longer time-scales.

### (c) Hierarchical structure of dominance

It should be noted that there is a variety of different pecking combinations which leads to deviations in the continuous sequence of the peck order … . Quite frequently one finds triangles, squares, etc.

—Schjelderup-Ebbe 1922 [1, p. 38]

Much ongoing research is aimed at understanding variation in the structure of dominance hierarchies and the ways in which individuals act within the confines of this structure. In this section of the theme issue, contributions explore the causes and consequences of hierarchical structure and the behavioural processes that underlie this structure. McCowan *et al*. [33] review recent work on dominance certainty, an emerging network-based measure of the structure of dominance hierarchies that reflects the flow of information through a dominance network. They find that in an extensively studied captive population of rhesus macaques, both dominance rank and dominance certainty are related to health outcomes for individuals and to position within the broader multi-dimensional social landscape, emphasizing how properties of dominance networks can influence individual outcomes in ways not fully captured by rank. Redhead & Power [23] also take a social network perspective on the structure of dominance hierarchies, this time in human societies. They review the sociological literature and highlight feedback loops linking individual-level determinants of social status, the meso-level properties of social networks and the macro-level structure of resulting hierarchies. Like McCowan *et al.* [33], Redhead & Power [23] emphasize that a full understanding of dominance hierarchies requires viewing them as part of a multi-layer fabric of overlapping social networks. Boucherie *et al*. [74] examine hierarchical structures in wild and captive raven groups and find that, while wild groups have much more fluid group membership than the captive groups, linear dominance hierarchies emerged in both. These results suggest that ravens are capable of maintaining many differentiated relationships in the face of considerable demographic change. The authors conclude with a discussion of how these open, fluid societies can favour advanced cognitive abilities for fine-tuning behaviour to the ever-changing social environment.

The final two contributions to this section of the theme issue focus on how behavioural processes operate within and give rise to, structured social hierarchies. Dehnen *et al*. [75] explore differences in the use of high- and low-cost aggression in vulturine guineafowl, a gallinaceous bird with steep and linear dominance hierarchies. They find that high-cost aggression is preferentially directed towards competitors of similar rank, while low-cost aggression is directed towards group members further down the hierarchy. In line with recent work on aggression heuristics across species [40], these results reveal how strategic competitive decision-making by individuals structures the aggression networks underlying dominance hierarchies. Hamilton & Benincasa [76] take a modelling approach to explore the underlying processes that give rise to size-structured dominance hierarchies. Inspired by the biology of social teleost fishes, they implement two models examining the structural effects of size-based competition outcomes and growth suppression. These models suggest that strategic investment in growth suppression of similarly sized individuals can produce the stable size-structured hierarchies found in nature. Complementing the results of Dehnen *et al.* [75], the authors find that costly investment in growth suppression was favoured in cases where competitors were of similar sizes.

### (d) Dynamics of dominance

The peck order in a flock of chickens can change over a period of time … if one hen rebels against her despot

—Schjelderup-Ebbe 1922 [1, p. 43]

To understand the structure, function and evolution of dominance hierarchies, it is crucial to understand how they emerge and change over time. In this section of the theme issue, contributions take a dynamic view of dominance hierarchies to advance insight into the function of these structures. Tibbetts *et al*. [57] examine the mechanisms underlying the formation and maintenance of hierarchies. In their extensive review, the authors find that these two dynamic processes are underpinned by separate sets of behavioural mechanisms and signals. They conclude by highlighting opportunities for future insight, such as the use of network analysis to better understand the interaction between individual attributes and self-organizational processes during the establishment of rank, the causes and consequences of hierarchy instability, and the cognitive mechanics underlying dominance hierarchies in different species. In line with this last suggestion, Wallace *et al.* [77] use a social perturbation experiment to explore how behaviour, physiology and cognition change alongside fluctuations in dominance rank in a species of social cichlid fish. They find that fish undergoing social ascent in response to the perturbation changed in multiple physiological and cognitive phenotypes. Using a principal components analysis assaying a suite of cognitive and physiological attributes before the perturbation treatment, the authors identified features of fish that predispose them to social ascent in response to the experimental perturbation. Finally, Strauss & Shizuka [78] explore open questions in the study of the dynamics of dominance hierarchies, describing how hierarchy dynamics can be understood at three organizational scales—individual, dyadic and group. The authors highlight five open questions about dynamics operating at these different scales. Although challenges exist to addressing these questions, the authors suggest some solutions to these challenges.

## 4. Conclusion

Every bird is a personality. However, the word ‘personality’ must not be understood to have the same meaning in this statement as when used in regard to human beings. What is meant here is that any one bird, irrespective of the species to which it belongs, is absolutely distinct in character and in the manifestations of character from any other bird of its species.

This may sound odd, but that is only because the individual and social psychology of birds has been regarded too superficially. No attempt has been made to know each individual bird in a given flock. So to know them, however, is the most important prerequisite for the full understanding of the general and comparative psychology of birds.

—Schjelderup-Ebbe 1935 [4, p. 947]

The key to Schjelderup-Ebbe's insight into hierarchical structure of animal societies was his view of animals as unique individuals rather than as instantiations of an essential species prototype [1,4]. This view, enabled by the identification of individuals based on their unique physical features, unlocked for Schjelderup-Ebbe the ability to detect and measure the profound effects of the social environment on the behaviour and experience of individuals. Subsequent work in many species has since revealed how the apparent anonymity of a flock of indistinguishable birds belies what is in fact a rich assemblage of diverse individuals varying in multiple dimensions, including their tendency to dominate others. This study of intraspecific variation in behavioural tendency, social phenotypes and fitness outcomes is now a central maxim of behavioural ecology, and advancing this perspective may have been Schjelderup-Ebbe's greatest contribution to the field.

A century of research has situated dominance as a central concept in the study of animal behaviour, ecology and evolution. Over these years, a number of features of dominance hierarchies have been established through careful research and vigorous debate. Much of the progress over the last century has confirmed, quantified or specified some of the intuition shared by Schjelderup-Ebbe in his foundational paper [1].

Despite this considerable progress, there remain a number of areas where our understanding of dominance hierarchies continues to grow, and these areas are highlighted in the contributions to this theme issue. A few cross-cutting themes emerge from the contributions to this theme issue as vibrant areas of ongoing or future progress, supporting the suggestion by Hobson [5] that we may be entering a more cross-disciplinary phase in the study of dominance hierarchies. Many contributions highlight the rapid development of advanced quantitative tools for social network analysis as playing an important role in driving new insight into the structure and function of dominance hierarchies (Tibbetts *et al*. [57], Shimoji & Dobata [71], Redhead & Power [23] and McCowan *et al*. [33]). Multiple contributions also prompt more work into the intersection between cognition and dominance (Milewski *et al*. [70], Dwortz *et al.* [72], Boucherie *et al*. [74], Wallace *et al*. [77], Tibbets *et al*. [57] and Strauss & Shizuka [78]), for instance by exploring the cognitive underpinnings of dominance relationships. Another emerging topic in this theme issue is the utility of rank manipulation experiments for shedding new light on dominance hierarchies (Simons *et al*. [68], Wallace *et al*. [77], Strauss & Shizuka [78] and Milewski *et al*. [70]), particularly for deciphering causal relationships from correlates of dominance status. Finally, in including targeted experiments (Simons *et al*. [68] and Wallace *et al*. [77]), observational studies of captive groups (Knoch *et al*. [73], McCowan *et al*. [33] and Boucherie *et al*. [74]), long-term individual-based observational studies (Anderson *et al*. [67] and Dehnen *et al*. [75]), comparative approaches (Strauss *et al*. [22]), theoretical frameworks (Lewis [69], Zeng *et al*. [24] and Strauss & Shizuka [78]) and modelling (Hamilton & Benincasa [76]), the theme issue highlights how research into dominance hierarchies uses the full breadth of tools available to modern biologists.

The breadth and depth of the contributions to this theme issue speak to the continued utility of dominance hierarchy research in driving advances in the evolution and ecology of social systems. Reflecting on the last 100 years of dominance research reveals how Schjelderup-Ebbe's early ideas and the subsequent research helped drive a shift from an essentialist view of species characteristics to the modern recognition of rich inter-individual variation in social, behavioural, and physiological phenotypes.

## Data accessibility

This article has no additional data.
